# Hospital and Institutional News

**Published:** 1915-04-17

**Authors:** 


### HOSPITAL AND INSTITUTIONAL NEWS.

### SIR ALFRED KEOGH AND THE NEED FOR DOCTORS.

Particular interest attaches to the publication of the statement made by Sir Alfred Keogh, Director-General of the Army Medical Service, at the recent conference with a special committee of the British Medical Association with a view to securing a larger number of doctors for the Army. The question resolves itself into how the civil profession can best help to provide whole-time medical men ready to serve abroad, and civil doctors willing to help in their own districts at training depôts, camps, or hospitals. Sir Alfred's suggestions of the way in which the civil practitioner could best help were five in number. He urged first that all medical men under forty fit for active service should apply for temporary commissions in the R.A.M.C., making a special appeal to young general practitioners in this direction, and pointing out the vacancies existing for various specialised branches of medical work. Secondly, he pointed out that doctors over forty are not being sent abroad at present, though they may be later, but are eligible for commissions and much required. Thirdly, doctors are wanted who can give a fixed time and so many hours each day to such military work as reporting sick with minor ailments, and looking to camp, barrack, and billet sanitation. Fourthly, doctors are required who cannot give specific time, but would be willing to attend the dependants of officers and soldiers at good rates of pay. Lastly, doctors are wanted who could arrange to help neighbouring doctors engaged in military duties, under terms to be settled by mutual agreement. The committee of the British Medical Association at once drew up a letter asking the divisions at home to hold early meetings and consider the above appeal. The rate of remuneration, it will be recalled, is 24s. a day, inclusive of all money allowances except travelling allowance and expenses when travelling on duty, an allowance of £30 for uniform, and a gratuity of £60 at the termination of service if this has proved satisfactory.

### "THE HOSPITAL'S" PLEA FOR PART-TIME SERVICE.

The above suggestions are encouraging in that they show that the authorities are beginning to recognise that part-time duty from civil practitioners who retain their civil status must no longer be rejected. We pointed out in The Hospital of March 27 that the solution of the present scarcity of doctors (not only, it must be remembered, for the needs of the Army, but also for the needs of the civil population) lay in making use of part-time service. We also showed that to appeal for suitable volunteers must largely be useless, since the very great majority of those who are suitable have already applied for commissions. We indicated that for domestic and other reasons it is impossible for many practitioners to give up their means of livelihood on the strength of an appointment which cannot be permanent; to say nothing of the pressing claims of hospital appointments which must after all be carried on. We added in so many words that "there is no doubt that many members of the profession would find, say, two hours a day for military duty in some form or other, provided that they are left free otherwise to continue their civilian work." The official adoption of this attitude is welcome, and should help the solution of the difficulty, since it is really from the adoption of the part-time system, as outlined in Sir Alfred Keogh's last three suggestions, that relief must come.

### PANEL CHEMISTS AND THEIR ACCOUNTS.

Good news for panel chemists was communicated at this month's meeting of the Council of the Pharmaceutical Society, when the secretary reported upon an interview which a deputation from the Society had last week with Mr. E. S. Montagu, Chairman of the Insurance Joint-Committee, on the question of the delay in the payment of chemists' accounts and the defects in the Drug Fund in many districts. Panel chemists have had a very real grievance in that they have had to wait for long periods for the settlement of their accounts; in many areas the chemists have had a still greater grievance in that their bills have been discounted, in one or two cases to the extent of 30 to 40 per cent., because the sums allotted for the provision of drugs, etc., has been to that extent insufficient. The chemists, whose chief spokesman was Mr. Edmund White, President of the Pharmaceutical Society, made out a very good case, and Mr. Montagu met them in a reasonable spirit. He promised that there should be a more prompt settlement of accounts, and that a sum of £60,000 which had been granted by Parliament should be made available for reducing the discount on the bills for 1913 and 1914. Thus the chemists in areas where the accounts have been heavily discounted will receive a good proportion of the amounts which some of them had almost written off as bad debts. Mr. Montagu clearly stated that this arrangement was not to be regarded as a precedent, and it is to be hoped that in future years there will be no necessity for such an emergency arrangement. In a proper businesslike affair there should be no question of the discounting of bills; and with the exercise of due economy in prescribing and other adjustments both ends must be made to meet.

### VACANT MEDICAL POSTS NOT FILLED.

It is very plain from reports which have reached us that pressure is being put upon local authorities to postpone the making of any appointments which would be likely to command the services of medical men fit to take up commissions in the Royal Army Medical Corps. As a consequence of this pressure several of the vacancies announced in the medical journals quite recently have not been filled. The specific instances which have come to our knowledge have been appointments relating to school medical inspection and to tuberculosis work. Such action on the part of local authorities is readily to be understood when due consideration is given to the recent pronouncement of Sir Alfred Keogh, and it may be confidently expected that for the present those who have the power to make these appointments will hold their hands. In point of fact, the present holders of appointments such as those referred to have already had it suggested to them that their work might be held up for a year or so without much disadvantage to the community, while the officers concerned would be free for military work. This attitude is, however, by no means general.

### HOSPITAL AUTHORITIES AND INAPPROPRIATE ADVERTISEMENTS.

Signs are not wanting, nevertheless, that a more correlated action by local authorities will presently be called for. A survey of the advertisements in the principal medical periodicals discloses on the one hand the increasing dearth of resident medical officers for hospitals, and on the other the as yet unrestrained competition by various authorities for school doctors, assistant medical officers of health, and the like. As an example of the former circumstance may be mentioned the throwing open to women of appointments at the Middlesex Hospital as house physicians. In regard to the second class of appointments one cannot fail to note the diversity of the conditions; some authorities have evidently begun to realise the present state of affairs, and have widened their portals; others have apparently not acted in the same spirit—in fact, one appointment of an important character is only open to candidates not over the age of thirty-five—a feature peculiarly inappropriate and unnecessary both for the post and the times.

### THE DIFFICULTIES DUE TO SMALL POOR-LAW AREAS.

Mr. H. R. Williams, the Local Government Board Inspector for North and South Wales and Monmouthshire, draws attention in his annual report to the difficulties in inducing Boards of Guardians to combine to carry out schemes of classification. He says: " In most of the thirteen counties in this district it would be possible and advantageous for the inmates now to be classified in separate institutions, and by mutual interchange the existing buildings could be devised and modified to accommodate the entire number of inmates without much additional expenditure; whereas if the requisite classification had to be carried out by some of the small Unions ' on their own ' it would be impossible to do so without involving a very heavy burden on the ratepayers. There has been considerable difficulty in making progress owing to the fear which obsesses some Guardians that their authority is, to some extent, to be curtailed, or that the project may result in some disadvantage to their particular Union." As an instance of the difficulty experienced he cites the case of a county in North Wales with four Unions, where a scheme failed because the Guardians of the only Union with buildings suitable for housing lunatics objected absolutely to their institution being converted into a " lunatic asylum." Another Union in South Wales refused to assent to the removal of their " lunatics " on account of the hardship of taking them away from their friends. On investigation Mr. Williams found that these inmates had no relatives or friends, and that the grievance was a purely imaginary one.

### THE EXAMPLE OF BIRMINGHAM.

As a contrast to this we may compare the results which have followed from the amalgamation three years ago of the three Poor-Law Unions in Birmingham. Before the amalgamation one of the Unions had decided to build a new infirmary at a cost of £100,000. By introducing a system of classification, and by utilising fully the institutions at its disposal, the Amalgamated Board found it unnecessary to proceed with the scheme. Amalgamation has led to efficiency, economy, and uniformity of administration. The new board has found it possible to make arrangements whereby those in their care can be placed in institutions specially adapted to their capacities and requirements. The whole administration has been reorganised, the scale of outdoor relief increased, and a revision committee appointed to investigate the circumstances of each inmate. During the three years nearly 40,000 cases have been considered by this committee, and in many instances inmates have been induced to make a fresh start as self-supporting persons. The value of uniformity of administration has been particularly marked in connection with the problem of vagrancy. The centralisation of the work has resulted in a decline in the number of vagrants from 29,823 in 1912 to 15,560 in 1914. A consideration of these results and of the difficulties in the way of voluntary combination leads irresistibly to the conclusion that the amalgamation of Unions by Local Government Board action is the line along which the Poor Law should progress in the immediate future. It is a step towards the goal to be achieved—the amalgamation of the Poor Law with the other public services under the control of one body.

### TRIBUTE TO THE EX-CHAIRMAN OF THE SATURDAY FUND.

The ex-chairman of the Council of the Hospital Saturday Fund, Mr. H. Gower, was the recipient of a well-deserved presentation at the annual general meeting last week. The Hon. Harry Lawson, as treasurer of the Fund, at the request of Mr. John Neal, chairman, presented Mr. Gower with an illuminated address, which formed an acknowledgment of his services from the members of the executive committee and a few friends. In making the presentation Mr. Lawson pointed out that Mr. Gower had acted as chairman for three exceptionally anxious years, and had been for twenty-three years a most assiduous and conscientious collector, and a member of the finance committee. The sums that he had collected, Mr. Lawson remarked, formed a testimony alike to his enthusiasm and his ability. It needs little emphasis to explain the critical circumstances which have surrounded the fund of recent years, when it is recalled that the income approached its highest point in 1911, when the operations of the Insurance Act supervened, so that during the latter half of 1912 and the whole of 1913 there was a definite financial set-back. Hardly, moreover, had there begun a recovery during 1914 than the war came, once again to create a time of difficulty. What the report describes as the keen competition of the various patriotic funds has curtailed the rate of increase. It is noteworthy, however, that the work of collecting and distributing has been carried on enthusiastically by a new batch of honorary collectors, and the disturbance created by the war has not been followed by any falling off of energy.

### THE "TIGERS'" BED AT LEICESTER.

Although all the fixtures of the Leicester Football Club were abandoned at the beginning of the season, it is gratifying to record that the club has been able to arrange its annual match for the Royal Infirmary. Difficulty was experienced by the honorary secretary, Mr. T. H. Crumbie, in securing a visiting team, but the well-known "Barbarians," composed largely of military players, kindly consented to form the opposition, and as a result it is hoped to add about £120 to the fund for the "Tigers'" Bed, now in process of endowment. The result is considerably less than usual, but the spirit of helpfulness on the part of committees and players is still active, and we record the effort as an example of what can be done by enthusiasm, even at a time of national crisis like the present.

### WOUNDED SOLDIERS IN POOR-LAW INSTITUTIONS.

Wandsworth Board of Guardians lately decided to anticipate any Government request for accommodation by offering to the War Office a portion of their institution at Swaffield Road for sick and wounded soldiers. The building will accommodate 120 patients, and is entirely cut off from the rest of the establishment. It has the added charm of being situated in a quiet part of the borough, where neither trams or 'buses pass along. Already the Board is receiving wounded soldiers at St. James's Infirmary, some twenty-nine having been admitted to the institution.

### SUMMER FROST-BITE—A FRENCH VIEW.

According to the Paris correspondent of the official organ of the American Medical Association, Dr. Temoin, of Bourges, recently made an interesting report to the Paris Academy of Medicine on cases of frost-bite from the trenches. Dr. Temoin is of opinion that many of the cases are not really cases of freezing, being produced less by the action of the cold than by the constriction of the feet in a shoe that is too tight, aided by the impairment of the circulation of the leg produced by the bands (puttees) around the calves worn by many of the French soldiers. Dampness aggravates the condition, producing a maceration of the tissues of the foot, and making the shoes shrink still further. The low temperature favours vaso-constriction and the circulation becomes constantly slower. In support of this view he cites the example of the Russian and German troops, who wear loose, waterproof boots, and among whom frozen feet are not prevalent. The Academy has brought Dr. Temoin's report to the notice of the French Minister of War. How much there is in the argument, which is well worth attention, will be tested by the amount of frost-bite experienced now that summer is in sight, although, of course, the effects of an impaired circulation will be less in warmer weather.

### THE SALARIES OF TUBERCULOSIS OFFICERS.

Dr. S. Nicol Galbraith, assistant medical officer to the Lambeth Tuberculosis Clinic, has been granted by the Lambeth Borough Council an increase of salary of £25 a year for two years, until his maximum has reached £400 per annum. In a letter addressed to the Council, Dr. Gilbraith points out that the British Medical Association recently issued a memorandum, approved by the Local Government Board, in which is emphasised the fact that tuberculosis officers, whose duties include the charge of clinical arrangements concerning tuberculosis cases and whose relation as regard such cases to the local profession would be that of consultant, should receive not less than £500 a year salary, exclusive of travelling, office, and clerical expenses. He did the entire work in connection with the diagnosis, treatment, and segregation of tuberculosis, dealt with through the central clinic or dispensary; the clerical work also now entirely devolved upon him; while the general practitioners of the borough continued to make increasing use of the facilities for consultation. In 1913 the total attendances of patients were 2,542, which had grown in 1914 to 7,175. He added that all tuberculosis medical officers at present appointed in the Metropolitan area were receiving the minimum salary, £500 per year, as recommended by the Local Government Board. Dr. Galbraith was appointed at a salary of £300 a year, which in April last year was increased to £350, and now the Council has decided to fix the maximum salary in Lambeth for the office at £400 a year.

### AN OVERWORKED DOCTOR.

The difficulties and overwork prevalent in many practices during peace have been exacerbated by the scarcity of doctors, as was borne out at an inquest held at Wandsworth last week on a child of two who died without being seen by the doctor, who did not arrive in time, in spite of three messages being sent for him. Dr. Thyne, divisional surgeon, remarked that when he received the message he had not had his clothes off for forty-two hours, and was thoroughly exhausted. During that night he was called to a confinement, and when the message came again on Monday morning his surgery was full of people and, having no assistant, he could not get out. In reply to the Coroner's statement that he ought to get one, Dr. Thyne remarked that it was a difficult matter as so many medical men had left the neighbourhood. He added that he could have done no good if he had gone, since he knew the child, who, as the *post-mortem* examination showed, died from heart failure consequent on double bronchial pneumonia. The Coroner said that if Dr. Thyne had attended the mother's anxiety would have been allayed, and that he might have let his other patients wait. Every sympathy will be felt with this point of view, but the plain fact is that no tact or consideration can enable a medical man to take charge of more than a certain number of patients. Indeed, in South London particularly the prevalence of disease has accompanied the scarcity of doctors, and with the still increasing demands of medical men for the Army it seems almost inevitable that civilians will have to suffer from the scarcity of general practitioners.

### WOUNDED FROM THE DARDANELLES.

Direct evidence of the fighting in the Dardanelles reached England last week with the arrival at Plymouth of the hospital ship *Plassy*, from which about eighty wounded or invalided men from the Mediterranean were landed. The majority were from the Dardanelles, and stated that the torpedo-boat destroyers, while under fire from the batteries, effected very gallant rescues of the crew of the *Irresistible* and the *Ocean*, after the latter had been struck by floating mines. It appears also from reports of their statements that the land fighting is of a stubborn and desperate character, owing to the house-to-house resistance and opportunities for ambush which the villages afford.

### AN ARCHITECT AS HOSPITAL SECRETARY.

The value of an *x*-ray installation to a cottage hospital has been well proved in the case of that at Tonbridge, by means of which many cases of bullet wounds have been examined since the war, which otherwise would have had to be sent elsewhere. The apparatus, which was a present from Sir David Salomons, has been accommodated on the plan designed by Mr. S. W. Little, the honorary architect, who is also the honorary secretary of the institution. The result has been the addition of three bedrooms and a boxroom as an additional floor over the main staircase, and all the *x*-ray examinations in the neighbourhood have taken place at the cottage hospital. Occasion was taken at the recent annual meeting to acknowledge Mr. Little's skill as an architect in relation to the provision of the extra rooms, and a cognate point of interest was raised in regard to a legacy which was expended in connection with the extension. A legacy of £200 was left by Miss Pierce to name a bed or beds in her memory, and the committee were fully under the impression that they were entitled to use this money in the building of permanent additions to the hospital. The committee, however, have since received communications from the Charity Commissioners objecting to the use of the Pierce legacy for this purpose, and requesting the committee at once to invest the sum of £200 in trust securities. Unless a way out of the difficulty is found—and the negotiations were still in progress at the date of the meeting—the committee, having spent the money, will be faced with the problem of raising a sum of £200 over and above the ordinary needs of the hospital. Thus the new *x*-ray department is important from more than one point of view, though the spending of a legacy, ear-marked as this apparently was, is obviously an error of judgment. We can only hope that the good work already achieved by the *x*-ray department will lead to local efforts being made to raise the £200 required for investment, which would be the most public-spirited way out of the difficulty.

### INSURED PERSONS IN POOR-LAW INFIRMARIES.

Efforts are being made by the Poor-Law authorities of the Metropolis to induce the Government to alter the existing arrangements affecting persons undergoing treatment in the infirmaries who are receiving benefits under the National Insurance Act. In the actual working of the Act, especially during the last few months, it has been found that there is a greatly increasing number of insured persons being treated in the infirmaries, and the authorities are of opinion that it was not the intention of the framers of the Act to allow these people to get benefits in the infirmaries and at the same time receive their allowances under the Act. There is another side to the picture, however, which even those responsible for the Act had in view when they decided that the sums allotted to the sick under the Act should not be touched by Poor-Law authorities. In many cases the benefits received by a patient are the sole support of his family while he is laid aside, and even when he is discharged from the institution it may be some weeks before he can go back to work, so that the amount due to him will come as a very timely aid in a period of convalescence. The question bristles with difficulties, which in some cases have been surmounted by the authorities asking the patients who are receiving insurance benefits to make a voluntary contribution, but no pressure has been brought to bear upon them, and even in the very rare instances where it has been used it has been severely condemned by the Insurance Commissioners.

### A REMARKABLE EXTENSION AT NOTTINGHAM.

Important negotiations have lately been in progress between the War Office authorities and the board of Nottingham General Hospital, with the result that temporary buildings for an additional 150 beds are being erected at once in the hospital's grounds for the further accommodation of sick and wounded soldiers. The extension is remarkable in several points. While the War Office is raising half the money for the new buildings the other half, of £1,500, is being generously provided by two gentlemen—Mr. W. G. Player gives £1,250 and Mr. James Forman £250. The equipment is to be furnished by the War Office, and the beds are to be maintained on terms to be arranged. By this means the number of beds for civilian patients is not to be reduced. The scheme provides for two wards to be built on the grass in front of the main block of the old hospital. Each ward will measure about 150 ft. by 33 ft., and be 10 ft. up to the eaves, and the cubic space per bed will be about 800 ft. It is hoped in two months to complete the buildings, which are on the general lines of those war hospitals already erected under Governmental sanction. The architects are Messrs. R. Evans and Son. Heating and lighting will be by steam and electricity respectively, and every effort is being made to complete them in the shortest possible time. The sub-committee in charge of the details consists of Mr. Frederick Acton, Mr. Player, and Mr. Forman. The beds in the two wards (there will be seventy-five in each) will be placed in three rows, and the ward unit will, of course, comprise kitchen, bathrooms, and lavatories. The buildings are to be externally of weather boarding, finished within with compo boarding.

### RELIEF HOSPITALS AND CONVALESCENT HOMES

The pressure on hospital accommodation is forcing us to do things which would not have been approved before urgency made them inevitable, of which the demand for short-time probationers is an example. But the relief hospital itself is somewhat a new institution, occupying as it does a sort of half-way house between the hospital itself and the convalescent home. Now that the winter is over the need is not so great, but it is still to be feared that the civilian poor have suffered from the loose classification which has led to so many light cases being drafted to hospitals whose equipment entitled them to receive the most serious cases. With the spring campaign the voluntary aid hospitals will take on a new importance, but had they been used more fully in the past the poor would not have had to endure as much as has been expected of them. The relief hospital, therefore, can play a more important part than is apparent, and if its establishment leads to a better classification of patients it will be a clear gain.

### AN OCTOGENARIAN'S GIFT.

The sum of £1,000 has been presented to the East Lancashire Royal Infirmary by Mr. Thomas Critchley, one of the oldest tradesmen in Blackburn, to commemorate his eightieth birthday. Mr. Critchley has always been interested in the institution, having acted for twenty years as one of its governors. He desires his gift to be used to endow a bed bearing his own and his wife's name. It is interesting to recall that he was the first president of the North-East Lancashire Chemists' Association, and therefore that by occupation as well as by public spirit he has been concerned in one of the departments of hospital work. We have already recorded the fact of a bed being endowed as a memorial to a fallen officer; and if other people would follow Mr. Critchley's example in endowing beds to commemorate important dates or anniversaries in their own lives also, the date would be marked for posterity and a public cause be powerfully helped.

### PAYING PATIENTS IN COTTAGE HOSPITALS.

Last year Horsham Cottage Hospital was faced with the need for extending its accommodation, and only the outbreak of war led to its extension being postponed. Another change, or rather experiment, was, however, undertaken—that, namely, of admitting paying patients, twenty-three of whom have been received. At the recent annual meeting it was reported that the experiment had proved a success, not only from the point of view of the patients, but also from that of the hospital, which had benefited by the fees paid, so that, in the words of the report, "The committee found themselves in the fortunate position of being able to spend money for the improvement of the hospital." At the request of the commandant of the local Red Cross Detachment it was decided to admit as probationers members who held Red Cross certificates, four of whom have taken advantage of the proposal, and are now helping to nurse in hospitals for the sick and wounded soldiers. The president, Mr. E. D. L. Harvey, expressed the hope that the committee would extend the facilities offered. The hospital has had to record the loss of its honorary secretary, Mr. A. O. Streatfeild, who resigned to take up work elsewhere, and his work has been undertaken by Mr. M. Dewing. Mr. Streatfeild had occupied the post for three years, and owing to Mr. Dewing's engagements it is necessary to appoint a permanent successor to this office. It is interesting to hear of the success of the plan for admitting private patients, and there is no doubt that where local conditions admit this has much to commend it, though it is unfortunate that they should be taken in at a time when there is a large waiting list owing to the postponement of the contemplated enlargement scheme.

### WINSLEY SANATORIUM AND THE NEW SCHEME.

The contemplated new scheme for the better management of Winsley Sanatorium, which is to be submitted for approval to the Charity Commissioners, continues to approach but slowly towards a satisfactory settlement. It will be recalled, from previous allusions in our columns, that the institution originally was intended to provide for consumptives from Bristol and the counties of Gloucestershire, Wiltshire, and Somersetshire. The future plan is for a sanatorium principally for Bristol, Bath, and Wiltshire. Questions arising out of the representation of the various interested parties have proved dfficult, since, while Bristol has the lion's share, the Wiltshire County Council is anxious to be more fully represented. The recent conference which was held at Bath, in the hope of arriving at a settlement which would be acceptable to all parties, while certain minor matters were adjusted, left the representation difficulty unsolved. The sanatorium possesses about 100 beds for tuberculosis cases. As we pointed out in a previous issue of The Hospital, the conversion of the sanatorium from a private charity into a municipal tuberculosis hospital, by which the Bristol Corporation were practically usurping the founders' rights, had led to endless difficulties with the medical board. The Corporation's responsibility has been heavy, and the institution has been described by medical authority as more resembling an expensive convalescent home than a scientific sanatorium. A settlement therefore, is well overdue.

### THE LEEDS EXTENSION.

THE General Infirmary, Leeds, is one of the institutions which foresaw a shortage of nurses, and on the outbreak of war decided to appoint immediately twenty additional probationers. The schemes of the committee in other directions, notably in regard to the extension, have had to be delayed owing to the insufficient means and income against which the institution has struggled for so long. Thus all that was originally planned is not being carried out, but the committee have felt justified in proceeding with the new theatres and the out-patients' block, while the enlargement of the nurses' home is already in hand. It is therefore hoped to see the nurses' home completed in the autumn, while the remainder of the buildings are due to be handed over in the earlier months of 1916. As to war work in the meantime, the infirmary had been requested before the war to provide 115 beds for soldiers, but nothing had actually been arranged. This request was, of course, repeated on the outbreak of war, and since then the number of beds for soldiers has risen to 135. In view of the work which the institution has performed and is performing, the people of Leeds, and those who are probably profiting by the activity of certain industries, might well come forward to aid the committee in their uphill task of completing the extension scheme.

### ALCOHOL IN PATENT MEDICINES.

A QUESTION arising out of the discussions on the proposed curtailment of the consumption of alcoholic beverages is that of the sale of patent medicines containing alcohol. There is undoubtedly on the market a large number of patent medicines which contain alcohol, but there are at the present time few which contain it in sufficient proportion to justify the supposition that the medicines may be used merely as substitutes for alcohol. Some years ago, when a youth appeared at a Lancashire police-court to answer a charge of drunkenness, it was positively proved that his condition was due to the effect of taking a proprietary medicine largely composed of spirit, but it is safe to assume that the Excise authorities would keep a sharp look-out for preparations of this kind should there be any cause to suspect that their sale was prevalent. In Canada and the United States, before the comparatively recent legislation affecting the sale of secret remedies was enforced, there was a very extensive demand, especially in temperance States, for a proprietary medicine which contained 25 per cent. of alcohol, while a well-known preparation of sarsaparilla contained 23 per cent. Both these products had, at any rate, a small sale in this country, but it is to be hoped that any attempt to increase the consumption of so-called "medicines" of this kind will be promptly checked.

### THE NEW COMMISSIONER FOR RED CROSS RELIEF IN SERBIA.

SIR RALPH PAGET has been appointed British Commissioner for Red Cross Relief Work in Serbia. He will carry on the work under the auspices of the joint committee of the British Red Cross Society and the Order of St. John. The need for additional Red Cross units in Serbia has been much emphasised of late, and at a recent conference held in Paris the whole question was gone into with a view to co-ordinating the efforts of all the agencies available. Those who were present included the chairman of the British Red Cross Committee, the Hon. Arthur Stanley, and representatives of the French Government, the American Red Cross Society, and the Rockefeller Institution, and a scheme was drawn up to supply Serbia's needs without the risk of overlapping. The response to recent appeals encourages the hope that the necessary money will be available, and the extent of the need and the gratitude aroused by previous efforts are well seen in the fact that a street in Uskub has been named after Lady Paget, the new Commissioner's wife, to commemorate her work in connection with one of the Red Cross hospitals. The ravages of disease in both the civil and military population have been very severe, but with the new scheme it is hoped to tackle the problem successfully. It will be recalled that Sir Ralph Paget has been an assistant Under-Secretary for Foreign Affairs since 1913, having previously held a variety of diplomatic appointments, including that of Minister Plenipotentiary at Belgrade.

### THIS WEEK'S DRUG MARKET.

THE upward movement in the prices of many drugs continues, and it is now no easy matter to obtain supplies of several synthetic products. Croton chloral hydrate, phenazone, guaiacol carbonate, acetanilide, phenacetin, and lactates are all advancing in price, and are likely to continue to advance. Salicylic acid and salicylate of soda have not advanced in price appreciably since last report, but acetyl-salicylic acid is again dearer, present quotations being ten or eleven times the price quoted before the war, with an almost certain prospect of further advances. Borax and boric acid are dearer. Very high prices are asked for cocaine, but the future of this drug appears to be uncertain. A further advance in the price of bromides would not come as a surprise. There is little change in the position of cod-liver oil. A very large business has been done in rectified spirit, and hospitals have been taking the precaution of increasing their stocks in case the contemplated increase in the duty on alcoholic beverages should, as has unfortunately been the case hitherto, involve an increase in the duty on alcohol used in the preparation of tinctures and other compounds used in medicines. Antimonial preparations are dearer. Carbolic acid continues to advance.

### TO OUR READERS.

CONTRIBUTIONS are specially invited from any of our readers to these columns. They should deal with topical subjects and news. They must be authenticated for the information of the Editor only. The minimum payment if published is 5s. There is no hard-and-fast rule as to space, but notes of about twenty lines in length are preferred.

